# Management of Menstrual and Gynecologic Concerns in Girls with Special Needs

**DOI:** 10.4274/jcrpe.galenos.2019.2019.S0174

**Published:** 2020-02-06

**Authors:** Özlem Dural, İnci Sema Taş, Süleyman Engin Akhan

**Affiliations:** 1 İstanbul University, İstanbul Faculty of Medicine, Department of Obstetrics and Gynecology, İstanbul, Turkey

**Keywords:** Adolescent, developmental disabilities, menstruation

## Abstract

For girls with physical and developmental disabilities and their families/caregivers, puberty and menstruation can present significant problems such as vulnerability, abuse risk, unintended pregnancies, difficulties with managing menstrual hygiene, abnormal uterine bleeding, dysmenorrhea, behavioral difficulties/mood concerns or changes in seizure pattern. Healthcare providers may have an important and positive impact for both the adolescents and their families/caregivers during this stage of life. Whether menstrual manipulation is indicated should be decided after a detailed history is taken from both the patient and the caregivers to determine the impact of current problems on quality of life. It should be explained that complete amenorrhea is difficult to achieve and realistic expectations should be addressed. The goals for the management of menstrual concerns should be a reduction in the amount and total days of menstrual flow, reduction of menstrual pain and suppression of ovulatory or cyclic symptoms, depending on each individual patient’s needs. Advantages and disadvantages of available treatment methods should also be discussed.

## Introduction

Adolescence, the period of transition between childhood and adulthood, can cause many difficulties for developmentally delayed adolescents and their families/caregivers due to hormonal changes, which result in menstruation and other reproductive health issues. These patients are also at high risk of sexual abuse and unwanted pregnancies (1). With the onset of pubertal changes, adolescents and the families start to have concerns about menstruation although most are able to manage these menstrual periods well without any intervention (2,3). Therefore prepubertal counselling is important but it should be noted that any intervention should be postponed until menarche, since it may affect the natural growth pattern of the adolescent and may compromise the diagnosis of genital tract malformations or reproductive endocrine pathologies (4). For most adolescents with developmental delay growth progresses according to normal growth curves, however precocious puberty may occur in cerebral palsy patients (5).

## Gynecologic Assessment

Communication with these adolescents may vary in effectiveness due to their mental status, hearing and communication function. Clinicians may need to communicate with them either with simple language or with illustrations. In addition writing, sign language or a translator may be useful or even a necessity.

The biggest assumption is to consider these patients as asexual. These adolescents usually have similar sexual thoughts, feelings and in some cases experiences as their peers. Therefore questions concerning sexuality and risky behavior should be asked of these patients, preferably alone, and the confidentiality of the dialogue should be established (6). History should be taken according to current symptoms, main concerns and expectations from treatment. The impact of menstrual cycles on the health and hygiene of the adolescent should be discussed. Counseling should be given to both the adolescent and the family/caregiver regarding sexuality, sexual abuse, problems concerning menstrual cycles, contraception and need for menstrual suppression.

Since it is now advised to start taking the Papanicolaou (PAP)-smears after the age of 21 and sexually transmitted diseases can be screened with non-invasive tests, gynecologic examinations are now needed less (7). For inspection of the external genitalia, a “frog-leg” position is usually preferred. If the abduction of the legs is compromised, the patient should lie on one side and the legs may be elevated for the inspection. When gynecologic examination is indicated but cannot be performed due to lack of communication or lack of mobility, it should be carried out under sedation or general anesthesia. When a PAP-smear test is indicated but a speculum examination cannot be performed, the PAP-smear may be taken with palpation of the cervix and by guidance using the fingers. HPV vaccination is strongly advised for these patients between the ages of 9-26 (8).

## Sexual Education and Prevention of Sexual Abuse

Parents/caregivers of adolescents with special needs are often worried that their children constitute a high risk group for sexual abuse and unwanted pregnancies (1). These patients may not be able to differentiate between good intentioned acts of help and inappropriate behavior, since they are used to getting help for daily activities. In addition, since these patients are often considered asexual, they do not receive the same degree of counseling on these important topics as their peers. Thus it becomes the task of the clinician to provide additional, appropriate and sufficient sexual education for this group, as part of general health services, listen to the concerns of the families and consult with them on how to educate their children in these matters.

The healthcare provider should determine if an adolescent is safe and if he/she is capable of giving consent to any sexual activity. This requires an assessment of his/her level of understanding on these matters. Behavioral changes such as regression, social withdrawal or self-harm can be an indicator of abuse for these patients. Also during physical examination, pain and bruises in unexpected areas of the body and genital complaints such as discharge or pruritus should be considered as alarming signs (9). It is especially important that patients of age 12-14 years and older should be examined in complete privacy, if communication between the clinician and the patient can be established.

Sexual education should start by explaining basic aspects of sexuality, including anatomical features of both genders, and defining appropriate and inappropriate behaviors in both public and private places. The education should be continued with topics including sexuality, pregnancy, contraception and sexually transmitted diseases. A key subject in this education should be prevention of sexual abuse. Depending on the level of understanding of the patient, a basic education of NO-GO-TELL strategy must be given (9). This strategy teaches the adolescent to say “No” if he/she is not comfortable, to get away from that situation and to tell someone he/she trusts about what has happened.

## Menstrual Concerns

Menstruation can cause significant challenges to these adolescents and their families/caregivers. These problems include personal hygiene, irregular or heavy bleeding that is usually seen within 2-5 years of menarche, dysmenorrhea, mood swings, and medical problems exacerbated by menstrual cycle, such as menstrual migraine or catamenial epilepsy. Additionally, sexual abuse and unwanted pregnancies are also crucial concerns at this stage.

## Menstrual Manipulation

A detailed history taken from both the patient and the caregivers, including the problems caused by menstruation and its impact on quality of life, should be the first step in management. As in normal practice with the general population, any other factors causing these complaints should be ruled out (10). Some of the conditions that may cause menstrual disorders in this group of patient include increased incidence of hypothyroidism in Down Syndrome, drug-induced hyperprolactinemia in patients receiving antipsychotic medication, and increased frequency of polycystic ovary syndrome in epilepsy patients (11,12).

After determining the extent and cause of menstrual problems, clinicians should decide if menstrual manipulation is indicated and the possible treatment options, advantages and disadvantages of the available options and possible clinical outcomes of these treatments, all of which should be discussed with the patient and the family (Table 1). It should be explained that complete amenorrhea is difficult to achieve and realistic expectations should be explained. The clinical aim of menstrual suppression is to shorten the time of bleeding and decrease the menstrual flow (13).

**Nonsteroidal anti-inflammatory drugs:** Nonsteroidal anti-inflammatory drugs (NSAIDs), when used according the patients weight, may decrease menstrual bleeding volume by up to 30-40% in ovulatory cycles (14,15). This treatment option may be preferred in the management of heavy menstrual bleeding and dysmenorrhea.

**Estrogen containing treatment options:** Combined oral contraceptives (COCs) can be used continuously for menstrual suppression. This treatment achieves up to 50% amenorrhea, although spotting is commonly seen, especially at the beginning of the treatment. Tablets can be powdered if they cannot be swallowed whole, although they are generally small in size. In the general population, the risk of venous thromboembolism (VTE) for adolescents and young women is very low. Use of COCs doubles the risk, but the risk of VTE remains quite low in this age group (16). Combined contraceptives may also be used in the forms of dermal patch or vaginal ring. Even though patches are more effective in increasing blood estrogen concentrations, there is insufficient evidence to demonstrate whether this may lead to an increased risk of VTE or not. Still, it is considered logical not to offer this treatment option in immobilized patients (17). In addition the vaginal ring is not a suitable option for patients with restricted mobility.

Data concerning the usage of estrogen-containing methods in patients with restricted mobility or who are immobilized and its relationship with VTE are inconclusive. However, it should be noted that third generation COCs are associated with higher risks of VTE and thus are not advisable as first line therapy for these patients (18,19).

## Progestin-only Treatment Options

**1. Oral progesterone:** Cyclic use of progesterone can decrease the amount of bleeding in anovulatory cycles (20). In addition, daily progesterone use, including progestin-only oral contraceptives, can be used for menstrual suppression. Though amenorrhea achievement rates are low, its efficacy is also closely related to regular use.

**2. Depot medroxyprogesterone acetate (DMPA): **Amenorrhea can be achieved in approximately 90% of patients following the fourth dose of intramuscular DMPA injection, used at intervals of ninety days. Spotting may be seen, especially in the first three months. The biggest concern about this medication is the loss of bone mineral density and risk of fracture. However, this effect is transient and bone mineral density increases again upon discontinuation of the medication and routine follow-up of bone mineral density is not recommended in these patients, but adequate intake of calcium and vitamin D should be encouraged (21). Developmentally delayed adolescents are already predisposed to poor bone health, due to anticonvulsant medication use, reduced mobility, and undernourishment. In adolescents, if DMPA is the method of choice for menstrual suppression, it is important to re-evaluate whether to continue treatment yearly and to discuss the risks and benefits with patients and families prior to starting therapy and regularly thereafter at follow-up appointments (22,23).

A further concern with the use of DMPA is weight gain. Especially in the immobile population, even a small weight gain can impact negatively on independent functioning of the patient. Therefore changes in weight in patients using DMPA should be carefully monitored.

**3. Progesterone-releasing intrauterine device (IUD):** Even though spotting is often seen during the first months following insertion, amenorrhea achievement rates are high with this treatment option over the long term. Progesterone-releasing IUDs reduce menstrual flow and dysmenorrhea, even if amenorrhea is not achieved. Amenorrhea rates are higher with IUDs containing 52 mg levenorgestrel and its efficacy continues for around five years. Usually the insertion is performed under sedation or general anesthesia due to communication problems and mobility restrictions. Several studies investigating the use of progesterone-releasing IUDs in adolescents with developmental disability have reported amenorrhea rates of up to 70% and low expulsion rates and removal rates due to bleeding or pain (4,24,25,26).

**Other treatment options:** Subcutaneous implants are not suggested as first line therapy since it usually causes irregular bleeding and the amenorrhea rate is only about 20% (8,27). It is also a disadvantage that the insertion and removal will most likely need to be done under anesthesia. Endometrial ablation is also not recommended as a first line therapy, since there are no data on its use in adolescents and the amenorrhea rates are low in a young population. Even though some families request hysterectomy, since it is an irreversible technique with possible surgical risks and complications, it is not recommended unless there are additional medical indications present.

Premenarchal intervention is not suggested, since it may affect the natural growth pattern of the adolescent and may compromise the diagnosis of genital tract malformations or reproductive endocrine pathologies (4). It should also be remembered that most of these adolescents will tolerate menstruation well (4,9).

## Menstrual Mood Disorders

Premenstrual syndrome (PMS) is defined as the emotional and physical symptoms that are only seen in the luteal phase of the menstrual cycle (28). Although there is not enough data about the incidence of PMS among developmentally delayed adolescents, PMS has been reported in approximately 18% of adults with developmental delay (29). Most of these cases have shown a response with pain medication, suggesting that dysmenorrhea may be the cause of these menstrual mood disorders, especially in a population that cannot communicate easily. Diagnosis may be made by showing that these symptoms are cyclic and persist for at least 2-3 months. The first treatment option is NSAIDs. In cases with no response to NSAIDs, hormonal suppression with COCs, especially those containing drospirenone 3 mg/ethinyl estradiol 20 µg (24/4 regimen), or DMPA is widely used for the treatment of PMS. As an alternative option, or an additional approach for cases resistant to the first and second line therapies, selective serotonin reuptake inhibitors may also be used (30,31).

## Problems Concerning Epiletic Adolescents

Epilepsy is seen in nearly 10-20% of patients with cognitive disabilities and 30% of them have catamenial epilepsy. Catamenial epilepsy is defined as the epileptic seizure that occurs during menstruation or change in seizure frequency according to the menstrual cycle. Seizure frequency may increase during three phases. These are the periovulatory phase, the premenstrual phase or the luteal phase, when progesterone levels remains low in anovulatory patients. The increased frequency of seizures is related with increased estrogen/progesterone ratios. Estrogen acts as a proconvulsant whereas progesterone increases the seizure threshold. Although the data is inconclusive, progesterone use in the luteal phase or DMPA use has been shown to decrease seizure frequency in these patients (32). Also use of continuous COCs is thought to decrease the frequency of the seizures by achieving amenorrhea, but again there is inadequate data to reach a robust conclusion.

Some antiepileptic drugs may decrease the efficacy of hormonal methods by affecting with hepatic cytochrome p450 (33). In the presence of persistent breakthrough bleeding, the dosage of estrogen/progesterone should be increased or DMPA should be injected on a more frequent basis. Blood concentrations of Lamotrigine may be lower in patients using COCs, therefore monitoring should occur and doses should be adjusted accordingly (33).

## Conclusion

Puberty and menstruation is often complex for girls with physical and developmental disabilities and their families/caregivers. While premenarcheal counseling provides great benefits in the management of these patients, no intervention or medical treatment is recommended during this period. Menstrual problems affecting quality of life in post-menarche period can be managed successfully by using various hormonal methods. A clear explanation of the realistic expectations from the treatment and the advantages and disadvantages of the existing methods increase the success and continuity of the treatment.

## Figures and Tables

**Table 1:**
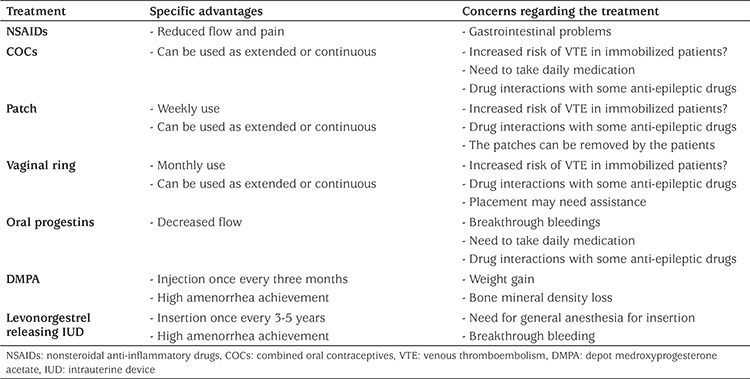
Treatment methods and their advantages and disadvantages
